# Sexual and reproductive health of Syrian refugee adolescent girls: a qualitative study using focus group discussions in an urban setting in Lebanon

**DOI:** 10.1186/s12978-021-01178-9

**Published:** 2021-06-24

**Authors:** Rayan Korri, Sabine Hess, Guenter Froeschl, Olena Ivanova

**Affiliations:** 1 Munich Medical Research School (MMRS), Medical Faculty of the University of Munich (LMU), 80802 Munich, Germany; 2 Department of Cultural Anthropology/European Ethnology, University of Göttingen, 37073 Göttingen, Germany; 3 Division of Infectious Diseases and Tropical Medicine, Medical Centre of the University of Munich (LMU), 80802 Munich, Germany; 4 German Center for Infection Research (DZIF), Partner Site Munich, 80802 Munich, Germany

**Keywords:** Adolescent girls, Refugee, Sexual and reproductive health, Urban setting, Syria, Lebanon, Qualitative research, Focus group discussions

## Abstract

**Background:**

The war in Syria caused the forced displacement of millions of Syrians to neighboring countries. Lebanon is the host country with the largest overall number of Syrian refugees per capita. Adolescent refugee girls experience a unique level of vulnerability during human emergencies and are at increased risk of suffering from poor sexual and reproductive health (SRH) outcomes. We conducted an exploratory qualitative study to learn about the SRH perceptions and experiences of refugee adolescent girls living in Bourj Hammoud, an urban setting in Lebanon.

**Methods:**

We employed a qualitative design with eight focus group discussions (FGDs) conducted with 40 Syrian Arab and Syrian Kurdish adolescent girls between January and March 2020. Every FGD consisted of five participants aged 13 to 17 years. A semi-structured guide was used covering multiple themes: menstruation, puberty, SRH awareness, and sexual harassment. FGDs were transcribed and analyzed using thematic analysis.

**Findings:**

The participants discussed adolescent girls’ health and named six elements of good health, such as healthy activities and self-protection. The majority of the FGD participants reported a lack of awareness about menstruation when they experienced it for the first time and the social stigma associated with menstruation. When defining puberty, they indicated its social link to a girl’s readiness for marriage and her need to become cautious about sexual harassment. Most FGD participants had very poor knowledge of the female reproductive system. Mothers were the most approached persons to receive information on SRH issues; however, the girls indicated a wish to receive advice from specialists in a comfortable and private atmosphere. All the girls reported that either they themselves, or an acquaintance, had experienced some type of sexual harassment. The girls rarely reported those incidents due to fear of being blamed or subjected to mobility restrictions, or forced to drop out of school.

**Conclusions:**

The findings show the refugee girls need for satisfactory knowledge on SRH issues and interventions to prevent sexual and gender-based violence that take into consideration the complexity of urban settings.

**Supplementary Information:**

The online version contains supplementary material available at 10.1186/s12978-021-01178-9.

## Plain language summary

After almost 10 years of war, Syria’s neighboring countries are hosting millions of Syrians who were forcibly displaced. Most prominent among these countries is Lebanon. Adolescent refugee girls are exposed to precarious conditions, which make them more prone to sexual and reproductive health (SRH) problems. This qualitative study was performed in Bourj Hammoud, an urban setting in Lebanon, in order to explore Syrian refugee adolescent girls’ SRH perceptions and experiences. The agenda of the International Conference on Population and Development (ICPD) in addition to the Inter-Agency Field Manual on Reproductive Health in Humanitarian Settings (IAFM) and its Adolescent Sexual and Reproductive Health Toolkit for Humanitarian Settings issued by the Inter-Agency Working Group on Reproductive Health in Crises (IAWG) formed the framework of this study. Focus group discussions were performed with 40 Syrian Arab and Syrian Kurdish adolescent girls, each group consisting of five participants aged 13 to 17 years. Different themes were discussed within the groups including menstruation, puberty, and sexual harassment. The participants talked about the social stigma related to menstruation and the social link between puberty, a girl’s readiness for marriage, and her need to be careful about sexual harassment. Most of the girls had insufficient information about the female reproductive system. The girls consulted their mothers to learn about SRH issues; however, they expressed a wish to receive well-informed advice from specialists in a safe atmosphere. All the girls reported incidents of sexual harassment, which happened either to them or to other girls they know; however, they were discouraged to report them because they feared other consequences, such as being blamed or not being allowed to go to school anymore. The outcomes of the study show the girls’ urgent need to have adequate information about SRH issues and appropriate interventions to prevent sexual and gender-based violence within complex urban settings.

**Supplementary Information:**

The online version contains supplementary material available at 10.1186/s12978-021-01178-9.

## Background

Wars and forced displacement cause lives lost, poverty, disease transmission, and shortage of life-sustaining services (1). In 2019, 70.8 million individuals, including 25.9 million cross-border refugees, were forced to flee their homes from brutality, armed conflicts, and natural disasters (2, 3). About half of the refugees worldwide are girls and women, who may experience intensified vulnerability and human rights exploitation (4, 5). Due to forced displacement, adolescent girls may lose support from their family and social networks, and be exposed to stressful conditions and unsafe environments, such as extreme poverty, having to drop out of school, human trafficking, risky occupations, and abuse. During humanitarian crises, adolescent girls are more prone to adverse sexual and reproductive health (SRH) outcomes, such as sexual and gender-based violence (SGBV), unwanted pregnancy, HIV infection, maternal death, and child marriage (6–8). At present, three-fifths of total worldwide maternal deaths happen in humanitarian and emergency settings (8). It is estimated that more than 500 women and adolescent girls die daily due to complications related to pregnancy and childbirth in such settings (8). Adolescents’ poor SRH outcomes are frequently caused by a shortage of adequate SRH knowledge in addition to a dearth of accessible youth-friendly facilities that could provide SRH services and products (8, 9).

Currently, and after almost 10 years of continuous war in Syria, 5.6 million registered Syrian refugees live in Lebanon, Jordan, Iraq, Turkey, and North Africa. Lebanon is considered the host country with the largest overall number of Syrian refugees per capita (10). According to demographic data published by the United Nations High Commissioner for Refugees (UNHCR), 23% of registered Syrian refugees are females under 18 years of age (10). In 2013, the United Nations Population Fund (UNFPA) conducted a situation analysis of youths in Lebanon affected by the Syrian crisis, which found that Syrian youths had insufficient knowledge about different SRH issues. For instance, 85% of the participants did not know which part of a woman’s menstruation cycle corresponds to potential conception (11). Syrian adolescent girls in Lebanon report feelings of isolation, not being safe, and being in distress because of the possibility of being exposed to sexual harassment and violence (12). They are also more likely to be subjected to child marriage due to parental safety concerns, difficult economic situations, and interruption of school education (13).

Although over 60% of the world’s refugees live in urban areas, there is a shortage of information about their living conditions and daily experiences (8). Most Syrian refugees reside in Lebanon in informal tented settlements (ITSs), rural or urban settings, with the majority living in poor and overcrowded urban contexts; however, the Lebanon Crisis Response Plan (LCRP) mostly targets refugees living in ITSs (14). Refugee women and girls living in urban settings frequently suffer from unmet SRH needs. The limitation of funds for SRH services, the physical difficulty in accessing those services, the lack of privacy and confidentiality, and the sensitivity around SRH are some of the reasons for these unmet SRH needs (15). The impact of living in refugee urban communities on adolescent girls, which is often ignored, varies in particular from that of adolescent boys or adult women (12).

The SRH experiences and needs of girls and young women are neglected in research for many reasons. The sensitivity of these issues and the necessity to perform studies with minor participants in line with ethical standards are some of these reasons. Furthermore, SRH in humanitarian settings is frequently not seen as important as other life-saving factors including water, sanitation, shelter, and nutrition (16, 17). However, this has started to change in the past few years, which have seen research on adolescent refugee girls begin to expand. Such research provides data to the humanitarian sector on the distinct concerns and difficulties experienced by this vulnerable group and shows how profoundly their lives are being affected by the crisis (12). Previously, some studies have been done on the SRH status of Syrian adult women in Lebanon, but very few studies have explored the SRH of Syrian adolescent girls (18–20).

This exploratory study aims to examine the SRH perceptions and experiences of Arab and Kurdish Syrian refugee adolescent girls living in an urban setting in Lebanon. The framework of this study is based on the agenda of the International Conference on Population and Development (ICPD) in addition to the Inter-Agency Field Manual on Reproductive Health in Humanitarian Settings (IAFM) and its Adolescent Sexual and Reproductive Health Toolkit for Humanitarian Settings issued by the Inter-Agency Working Group on Reproductive Health in Crises (IAWG) (21–23). The study findings will not only inform the work of Lebanese and international organizations and institutions, but also add to the existing global literature on refugee adolescent girls’ SRH by expanding the research to the Extended Middle East and North Africa (EMENA) countries. Thus, this study provides an opportunity to contribute to refugee adolescent girls’ well-being, consider their SRH needs, and avert poor SRH outcomes.

## Methods

### Study setting

Bourj Hammoud is an industrial area in North-East Beirut, which is considered one of the most densely inhabited areas in the Middle East. Its history of hosting refugees goes back to the 1920s, when the survivors of the Armenian Genocide arrived after fleeing persecution by the Ottomans (24). Nowadays, the suburb mostly accommodates inhabitants with lower socio-economic status: Lebanese citizens, Syrian, Palestinian, and Iraqi refugees, in addition to migrant workers from Asian and African countries (25, 26). In recent years, the area became a place of residence for many Syrian refugees in Lebanon (27). According to UNHCR, 8,747 Syrian registered refugees live in Bourj Hammoud (28). Finding appropriate accommodation in that area is considered problematic due to the challenging living circumstances, such as absence of clean drinking water, secure electricity, robust infrastructure, and sufficient hygiene standards (12).

### Study design

The framework of this study builds on the principles and goals of the ICPD in addition to the IAFM and its Adolescent Sexual and Reproductive Health Toolkit for Humanitarian Settings delivered by IAWG (21–23). The ICPD’s Programme of Action, which was held in Cairo in 1994 and attended by 179 countries, not only firmly placed Reproductive Health and Rights on the international agenda but also presented a major development in considering SRH care and services fundamental for all married and unmarried individuals, including adolescents and youth (21). In the section VII of its Programme of Action, the ICPD determines SRH care for people of suitable age groups, which covers different services including: i) Information, education and counseling on human sexuality and reproductive health; ii) Prevention and surveillance of violence against women, care for survivors of violence and other actions to eliminate traditional harmful practices. Moreover, the ICPD highlights the particular challenges faced by migrants and displaced people in general, and women and adolescents specifically, when accessing SRH services and how these services should complement their precise SRH needs (21).

In its 2018 version of IAFM, IAWG acknowledges sexual and reproductive health and rights (SRHR) as a key element in accomplishing human rights goals such as the right to health, ill-treatment, privacy, and education, in addition to protection from discrimination, specifically that which is based on sex and gender. This version of IAFM underlines the importance of individuals affected by crises and living in humanitarian settings obtaining comprehensive SRH knowledge and services in order to improve SRHR worldwide. Additionally, the manual focuses on those individuals’ ability to make informed decisions regarding their SRH without being subjected to intimidation, intolerance, and violence (22). IAWG gives further explicit focus on adolescent sexual and reproductive health (ASRH) through a toolkit that guides IAFM. IAWG is a humanitarian mandate, in which the promotion and delivery of knowledge and accessible services are important components. On the one hand, the toolkit shows the potential harmful consequences if adolescent sexual and reproductive health and rights (ASRHR) in emergency settings continue to be overlooked. On the other hand, it draws attention to ASRHR and the positive health and societal outcomes of providing adequate ASRH information and services for refugee adolescents in general, and refugee adolescent girls in particular, in coordination with them (23).

Since the main aim of this study was to learn about refugee adolescent girls’ SRH perceptions and experiences, we employed a generic qualitative research approach (29–31). Focus group discussions (FGDs) were chosen for this study, as they give the opportunity for participants to share their knowledge, beliefs, and experiences within a discussion that is shaped by their own concerns and preferences (32, 33). The flexibility of this research method not only allows participants’ reflexivity during the description and interpretation of their own views and practices, but also flattens the power hierarchy between the participants on the one hand, and the researcher on the other hand, since the focus is on the participants’ thoughts and expressions (34–36). What makes FGDs a unique dialogical qualitative research method is the interaction between the participants, which allows both similar and diverse contributions to the discussion to develop, and a richer interpretation of these contributions (37, 38). FGDs were found to be a preferable research method for adolescents compared to interviews, since FGDs involve participants’ peers in an informal and less intimidating environment (39, 40). For comparing FGDs and centering them around important items, as mentioned above, we used a semi-structured guide with questions on specific themes: menstruation, puberty, SRH awareness, and sexual harassment. These health issues and SRH topics were chosen as the main themes to be examined, based on the tool validated by UNFPA and Save the Children: Adolescent Sexual and Reproductive Health Toolkit for Humanitarian Settings (41).

### Sample participants

Three Syrian female community gatekeepers from different age groups, various Syrian regions and ethnic backgrounds, and with distinct socio-economic characteristics (education, employment, and household income) were responsible for recruiting the participants to ensure an inclusive approach, guaranteeing the possible diversity in terms of age, social, ethnic, and religious background. Gatekeepers’ involvement in research on sensitive topics, such as SRH, is key to reach refugee groups who show concerns about trust (42–44). The first phase of field work, prior to conducting the FGDs, included developing relationships with local actors and inhabitants, in addition to identifying the area’s particular context (45, 46). The first author was introduced to the gatekeepers by a fellow researcher from Lebanon who had previous field work experience in Bourj Hammoud. The gatekeepers were able to link the first author with the participating girls’ families, which they knew prior to the study, due to the shared and tightly connected social networks among Syrian refugees in the area. Purposive sampling was used based on the characteristics of the participants: gender, age, nationality, and ethnicity. Sampling aimed to include married and unmarried Kurdish and Arab Syrian adolescent girls, aged between 13 and 17 years, and from different Syrian governorates. The sample size of the study, which was determined based on the principle of data saturation, consisted of 40 participants in eight FGDs. Every FGD was formed of five participants. Data saturation is achieved when no additional knowledge is produced from new FGDs (47, 48). In the case where information is being repeated, the researcher decides to stop data collection and move to data analysis since the representation of participants’ views has been adequately covered (49).

### Data collection

Data was collected from January to March 2020. Data collection was performed by the first author, a Lebanese female doctoral researcher, who is an Arabic native speaker, knowledgeable about the research context, and has previous experience in qualitative research.

Each FGD consisted of five participants aged between 13 and 17 years with a mixture of marital statuses. The community gatekeepers were responsible for arranging the groups under the direction of the doctoral researcher. Sociocultural homogeneity was ensured within every group, assigning participants who had the same ethnicity, came from nearby Syrian regions, and shared similar social networks to the same group. This homogeneity helped to create a feeling of comfort and familiarity among the participants, enabling a smooth flow in the discussions, and insuring active participation from all the girls (37, 39, 50).

FGDs were performed privately and without the presence of a parent or a caregiver. They took place in a closed room in the gatekeepers’ apartments or in one of the participants’ apartments, since these places were easy to access for all participants and were considered safe and trustworthy by the participants’ parents or caregivers. In addition to the researcher, who established and encouraged dynamic group interaction throughout the discussion, a student assistant was present to take notes on non-verbal communication. At the beginning of each FGD, the participants were asked about some demographic information and then were directed into the discussion through a semi-structured guide with questions on the specific themes. The FGD guide is presented in Additional file 1. Flexibility to add new topics throughout the discussions was possible until data saturation was achieved. The duration of the FGDs ranged between 45 and 75 min.

### Data analysis

FGDs were audio recorded, transcribed verbatim, and translated into English. Field notes taken after every FGD were also used during the analysis. To preserve confidentiality, participants’ names were removed during transcription and every participant was assigned a participant identification number. Descriptive data analysis was done using thematic analysis (51, 52). A pre-defined codebook of themes and sub-themes was generated based on the study’s frameworks, the existing international literature on ASRHR in humanitarian settings, and the FGD guide. New codes were added for the newly developed themes that arose from the FGD data. For instance, the following codes were chosen for the theme on menstruation experiences and management: first menstruation experience; lack of knowledge; negative reactions; new practices; change of daily routines; menstrual pad buying; information on cycle and tracking; source of information; misconceptions; explanations provided; negative emotions; and social stigma. A sample of the codebook’s schematic representation is shown in Additional file 2. As a first step, the coding was performed independently by two co-authors for each transcript through a close line-by-line reading. As a second step, these two co-authors engaged in discussions to classify and confirm the themes and sub-themes. No major disagreements over code or theme identification took place between the co-authors. The co-authors could not go back to the participants to validate the findings as the participants were not assigned direct identifiers. Furthermore, the participants’ parents or caregivers were anxious about sharing their address or contact details due to security concerns.

## Ethical considerations and researchers’ positionality

Before starting data collection, the researcher clarified to the participants and their parents or caregivers the aims and significance of research projects in general and, as well as the specific aims of this study: the identification of SRH perceptions and experiences of Syrian refugee adolescent girls living in Bourj Hammoud- to inform academics and practitioners working in the field. The participants were informed about their right to withdraw their participation at any time. The girls’ participation in the study depended on their willingness to take part in the FGDs, their oral and written Arabic informed assent, and the oral and written Arabic informed consent of their parents or caregivers. Participants’ mothers, mothers-in-law, and aunts provided the majority of the informed consent.

Even though FGD as a research method presents various advantages when collecting data on sensitive topics from young participants, the different ethical challenges should be taken into account. Care should be taken when conducting research with young individuals, since they may not be able to fully express composite perceptions and opinions within groups. Their presumed limited abilities and competences when articulating ideas, in contrary to adult participants, might be among the reasons (53, 54). Furthermore, although FGDs give young participants the opportunity to contribute within an engaging group setting where they can learn from the views and experiences of each other, this process can be counter-educational when misinformation is shared (55–57). In cases where the researcher suspected that misinformation was circulating, she held a 15-min informal conversation with the participating girls after the closure of the FGD, where she corrected and rediscussed information. Another ethical challenge when conducting FGDs with young girls experiencing certain vulnerability is the possibility of revealing sensitive and personal opinions and experiences that could distress the participants after data collection (58). This was particularly challenging when discussing the theme on sexual harassment. Although the researcher took several actions and measures before, during, and after conducting the FGDs to preserve confidentiality and ensure that no participants are unintentionally harmed, it is crucial to take into consideration the safety and well-being of the participants in every future research that tackles sensitive topics such as violence against women. To deal with this challenge, the female researcher who was also the facilitator, created an asserting, cooperative, and non-judgmental atmosphere when conducting FGDs and employed a FGD guide that allowed flexibility by mentioning the themes that should be addressed in every FGD rather than certain fixed questions. This gave the young girls agency in navigating the discussion in a way that made them feel comfortable when interacting and sharing, without being anxious about others’ judgment (59).

As researchers in the fields of public health, forced migration, and cultural anthropology, we are devoted to human rights, social justice, and societal development. We believe in the importance of research in raising awareness about the living conditions and experiences of refugee girls and women on the one hand, and in employing its findings into applied programs and policies on the other hand. Our biases and expectations were frequently openly discussed within the team in an attempt to assure objectivity while designing the study, conducting the FGDs, and analyzing the data. These biases and expectations were drawn based on reports and articles from other studies investigating the health status of refugee women living in EMENA in general and refugee girls living in Lebanon in particular. Since our research team members come from different backgrounds—Lebanese and European—and have different perspectives on the topic of conflict-affected adolescent girls’ SRH based on their various fields of academic research, we believe that neutrality when conducting this study was greatly enhanced. Furthermore, the linguistic and ethno-cultural background partially shared by our team’s Lebanese researcher and the study’s Syrian participants provided potential relational and emotional benefits for the girls and their parents or caregivers, helping them to feel empathized with and appreciated due to a common linguistic and cultural understanding (60).

## Findings

The demographic characteristics of participants and the themes that emerged from the FGDs are presented below. Six themes arose from the FGD analysis: 1) understanding adolescent girls’ good health; 2) menstruation experiences and management; 3) perceptions of puberty; 4) knowledge about the female reproductive system; 5) the need for accurate information on SRH topics; 6) sexual harassment experiences and handling mechanisms.

### Demographic characteristics of the participants

A total of 40 girls participated in the FGDs. The mean age of participants was 14.5 years with 85% (n = 34) of the girls being Syrian Arab and 15% (n = 6) Syrian Kurdish. The participants came from six different Syrian governorates, with the majority (67.6%, n = 27) arriving from Aleppo. The majority of the participants were single (87.5%, n = 35), whereas three girls were engaged, one girl was married, and one girl was divorced. Around three-quarters of the participants (72.5%, n = 29) had been living in Lebanon for more than five years. About 67.5% (n = 27) of the girls had completed at least primary- school education and 30% (n = 12) had completed middle- school education. A total of only 57.5% (n = 23) of girls were currently in school and 45% (n = 18) were participating in non-governmental organizations’ (NGOs) activities and training. The detailed demographic characteristics of the FGD participants are presented in Table 1.

**Table 1:** Demographic characteristics of the FGDs participants

	Number (*n* = 40)	Percentage (%)
Age		
13 Years	12	30
14 ears	12	30
15 Years	7	17.5
16 Years	4	10
17 Years	5	12.5
Ethnic Group		
Arabs	34	85
Kurds	6	15
Governates of Origin in Syria		
Aleppo	27	67.5
Raqqa	4	10
Idlib	4	10
Deir ez-Zor	2	5
Damascus	2	5
Hama	1	2.5
Duration of stay in Lebanon		
< 1 Year	1	2.5
1–5 Years	10	25
> 5 Years	29	72.5
Educational level		
Never attended school	1	2.5
Primary-school	27	67.5
Middle-school	12	30
Currently in school		
Yes	23	57.5
No	17	42.5
Participate in available NGOs activities/trainings		
Yes	18	45
No	22	55
Marital status		
Single	35	87.5
Engaged	3	7.5
Married Divorced	1 1	2.5 2.5

### Understanding adolescent girls’ good health

None of the participants was familiar with the term SRH. When being asked about it during the FGDs, the girls could neither define it nor name any ideas related to it. Therefore, the question was modified from their comprehension of adolescent girls’ SRH in particular to their comprehension of adolescent girls’ health in general. The FGD participants named six elements of good health for adolescent girls: healthy activities; hygienic habits; absence of diseases; nutritious diet; good emotional health status; and self-protection.

The girls considered basic physical activities and sports; sleeping sufficiently; drinking enough water; contributing to household chores; and preserving home tidiness as daily acts that contribute to their physical well-being. One participant stressed the importance of haircare and cutting it short because it influences the growth of a girl’s body:

*“We take care of our health by cutting our hair. I have heard that the length of a girl’s hair affects her body height. If the hair becomes too long and she doesn’t cut it, she will stop growing.”* (14 years old).

Hands washing before and after meals; showering daily; body hair removal; nail cleaning; teeth brushing; changing clothes and underwear daily; changing pads frequently; and external female genital organ hygiene were the habits the participants connected to good adolescent girls’ health. The girls also mentioned lack of illness as a key sign of wellness. Wellness also included the absence of pain, regular doctors’ check-ups, medication, having strong immunity, and hormonal balance:

*“A girl should visit doctors with different specializations. Even if she cannot afford it, she can go to doctors who have free clinics or free campaigns. Women should also have breast cancer screening in those campaigns.”* (14 years old).

The majority of participants mentioned the importance of having to a healthy diet in order to have physical well-being. One participant claimed that girls and women should have particularly nutritious meals compared to other family members to have supplementary support, since girls and women have many responsibilities. Additionally, two participants talked about the need for a girl’s body wellness and strength to be ready for pregnancy and carrying a healthy baby. Few participants pointed out the connection between emotional health and physical health and how the status of the first affects the second:

*“To be honest, when I am psychologically at ease, everything in me rests. When I am sad, everything in my body starts to hurt.”* (16 years old).

The adolescents emphasized the need for self-protection to maintain good health. This involved avoiding carrying heavy items; preventing physical wounds; defending oneself when being physically or sexually harassed; and not getting involved in romantic relationships. According to the participants, all these factors have negative effects on adolescent girls’ health.

### Menstruation experiences and management

Menstruation experiences and management was the theme that emerged from the FGDs on which participants elaborated the most. Five sub-themes were discussed by the girls: 1) first menstruation experiences; 2) knowledge about the menstrual cycle and sources of information; 3) menstruation hygiene management (MHM); 4) adapted routines during menstruation; and 5) social, psychological, and physical experiences.

#### First menstruation experiences

The majority of the FGD participants described their first menstruation experience as shocking and scary. One of the participants explained that she was familiar with the topic from overhearing the women in her family talking about it, while another participant said that her mother briefly mentioned it to her before her first experience:

*“I did not know about it when I had my period for the first time. I thought it was a kind of diarrhea. I did not feel anything on the first day. On the second day, when it became worse, I told my mother. She said that I was having my period.”* (14 years old).

*“I knew about it because I had heard my sister and my sister-in-law talking about it before. I had my periods six times, six months, and I did not tell anyone about it. I was buying the pads without anyone noticing, using my own savings.”* (16 years old).

Some of the participants talked about hiding their discovery from their parents for some time because of embarrassment and their lack of understanding about what was happening, whereas others shared it immediately with female family members in order to get help:

*“I was scared. I didn’t tell my parents on the first day. I was showering every now and then thinking that it would go away. It was so scary… something happening for the first time in my life.”* (15 years old).

The participants also talked about the difficulty they faced when trying to use disposable pads in an effective way for the first time since they had not received any previous instructions about how to use them:

*“I changed the pads more than five times per hour when I had my period for the first time. I was not using them in the right way. No one had taught me how to do so before. When I showed my mother the leaked blood, she told me that I was not using the pad correctly. She taught me how later.”* (13 years old).

*“I was scared during the first menstruation; I was in school and did not know how to use the pads.”* (17 years old).

#### Knowledge about the menstrual cycle and sources of information

All participants mentioned their mothers as the first person they approached when they had questions regarding menstruation. Other female relatives were also consulted by some of the participants. The girls talked about receiving additional information about menstruation through informal talks in school with female friends and teachers. A few girls had the opportunity to participate in social activities offered by a local NGO and scouts, where the topic of menstruation was explained. One participant talked about watching YouTube videos on menstruation symptoms and how to have a healthy routine during menstruation. Another participant mentioned the mosque as the place where she received information about the topic:

*“I used to go to a mosque. The teacher of the mosque was explaining menstruation to older girls. I used to listen to her but did not understand what she was talking about exactly.”* (14 years old).

Half of the girls participating in the FGDs could not explain the reasons behind menstruation. The rest of the girls discussed menstruation as a sign of puberty; personal maturity; hormonal change; and transition from childhood to womanhood. They frequently talked about menstruation as a mechanism to get rid of the toxic blood in their bodies and to prevent diseases. The girls also linked menstruation to awareness of and ability to get pregnant. The majority of the participants did not know how to track their menstrual cycle. Only one of the participants was able to clearly explain the menstrual cycle’s three phases and duration:

*“I know about this topic because of social activities in scouts. A girl has her period every month. I check the date when I got my period and add 21 days to that. Let’s say I had my period on Monday and it was over on Sunday, I add 21 days to this to know when I will have my next period.”* (15 years old).

Many myths about beneficial and harmful practices during menstruation exist. The girls pointed out some restrictions that should be followed during menstruation. Consuming certain foods and drinks like oil, starch, and hot drinks are encouraged during menstruation whereas consuming cold food and drinks are not allowed:

*“I hate menstruation during summer because I can’t eat ice-cream. People around me say that it is harmful then, like cold water and soft drinks. We have to drink anise tea and it is very hot for that.”* (13 years old).

One participant complained about not being allowed to take painkillers during menstruation or drink more water because of concerns about becoming infertile or developing ovarian cysts:

*“I am in so much pain when I have my period. I ask my mom to take me to the doctor to give me medicine. Our relatives tell my mom not to do so because then I will not be able to have kids when I’m married. Also, people told us that if a girl takes medicine, she will not have her period often. Others told us that if a girl drinks a lot of water, she will have water sacs on her ovaries (cysts filled with fluid).”* (17 years old).

#### Menstrual hygiene management (MHM)

All FGD participants reported using disposable pads that were always bought out off their parents’ personal income or their own savings. The participants’ individual monthly needs varied from one to three packets of pads per month with the costs varying from $1.- to $2.5 per packet. The pads were bought from local stores like supermarkets and $1 shops (stores that sell low-cost items for one US-Dollar or less).

The participants had access to cheap disposable pads but complained about their bad quality. The girls described their experiences using the pads as unpleasant, painful, and irritating. Some participants could change their pads only one to three times per day. The mothers of most of the girls were responsible for buying the pads for them, which made them uncomfortable since their mothers did not know what the most suitable type of pads for the girls was. A few participants were satisfied with the choice of their mothers. The husband of the only married participant in the FGDs was responsible for buying her the pads.

The majority of participants felt shy and avoided going into stores to buy pads where the sellers were males. They preferred to ask a family member in order to avoid social stigma:

*“I buy the pads for myself. I walk for 15 minutes to go to a shop where the seller is a woman. So, I walk all the way there just to buy from her.”* (17 years old).

*“I don’t like to buy the pads myself. Sometimes the person selling in the shop is a guy… whenever I see him, I return the pads and don’t buy them.”* (15 years old).

Only a few participants did not express discomfort when buying from male sellers:

*“I don’t get embarrassed when buying from a man because everyone knows that girls menstruate… it’s normal.”* (13 years old).

#### Adapted routines during menstruation

The participants identified new habits and practices that they followed during menstruation. Wearing dark colored, long clothes that cover helped the girls overcome the anxiety associated with period stains. The participants preferred to stay at home during their menstruation with minimal physical activity for different reasons, such as having easy access to the bathroom, resting when having severe pain and heavy bleeding, and avoiding the exhaustion when at school. One of the participants considered menstruation a monthly opportunity to be exempted from household chores and carrying heavy items whereas another participant complained of being obliged to carry on with household chores while experiencing menstrual pain:

*“Regardless of the pain, it is good that I can sit alone. No one bothers me. I can do whatever I want. I am not required to do anything. I don’t have to do the household work or hold anything heavy.”* (14 years old).

*“I have to continue the household work when I have my period, even while experiencing pain around my hips.”* (17 years old).

All girls mentioned body hair removal in general and pubic hair removal in particular as a monthly habit that should be performed right before or after menstrual bleeding. In addition to that, they considered having an extra shower at the end of menstrual bleeding necessary. The habits raised are important for the girls for hygienic and religious considerations.

#### Social, psychological, and physical experiences

All participants expressed negative emotions when talking about menstruation. They described it as a burden accompanied by physical pain, which makes them feel lonely, uncomfortable, worried, embarrassed, and nervous. Participants pointed out the social stigma associated with menstruation and explained how it is considered shameful and improper within their community. The girls spoke about their concerns of having their menstruation when being outside their homes, which subjects them to gossips and bullying, mainly because of the possibility of staining:

*“It is something that makes me shy. I feel embarrassed to say it (menstruation).”* (15 years old).

One of the participants disagreed with her peers and explained that menstruation should not be distressing since it is something natural that happens to all girls.

*“There is nothing embarrassing about it because every girl will have her period, not only us.”* (13 years old).

### Perceptions of puberty

The majority of the participants were able to explain what puberty is through the description of changes in physical and personal characteristics, and social considerations. A few participants could not describe any change connected to puberty and were only familiar with the term itself.

The girls talked about breast development; body hair growth; starting menstruation; acne; height increase; hips widening; and weight change as the physical signs of puberty. However, they were all not aware of these changes before experiencing them:

*“The changes started to happen when I was 11 years old. My breast started to become bigger. I thought that it happened because I swallowed olives. I found it weird. I was so embarrassed to ask my mom.”* (14 years old).

Most of the participants mentioned the need to accept the physical changes accompanying puberty although they found them repelling and distressing, since they expose them to embarrassing situations and bullying, especially from their male schoolmates and friends:

*“I find the changes ugly, but they will happen anyway. They happen for us in order to grow older.”* (14 years old).

The participants discussed puberty as a stage where girls leave behind childhood, and along with it play, to become mature and determined, with the ability to understand life and handle responsibilities. A few participants claimed that puberty means a girl’s need to become cautious about sexual harassment and others’ behaviors towards her. The FGD participants complained about suddenly being under ‘social surveillance’ once they reached puberty. They spoke about their parents’ new concerns and rules, which focused on girls’ modest clothing and ‘good behavior’:

*“It is something very annoying because parents start to control a lot. I could wear shorts when I was younger. They tell me that I’ve reached puberty and became old now, so I can’t do that anymore. Everyone watches whatever I do. As if they are waiting for a mistake.”* (15 years old).

Adolescents also pointed out the social association of puberty with readiness for marriage:

*“The first thing the moms say when we start puberty: you are suitable for marriage now.”* (15 years old).

The girls described their feelings of unhappiness at being treated unexpectedly as adults when they still wanted to live in their childhood:

*“As if there are two teams. One for kids and one for adults. You suddenly leave one and join the other. I did not like that. I don’t like the new team.”* (15 years old).

*“I wish I could go back and be a child.”* (14 years old).

In one of the FGDs, participants talked about traditions performed by their female relatives once they reach puberty in order to control the changes happening to the body and its development:

*“Once my breasts started to become bigger, my aunt brought a coffee cup and put it on my breast, so its shape would get formed like the cup and not become so big. She also took three of my fingers and immersed them in water and salt, to have my period for only three days.”* (17 years old).

### Knowledge about the female reproductive system

Most FGD participants had very poor knowledge about the female reproductive system. They confused it with digestive and urinary systems when asked about the location of the female reproductive system. Some participants were only familiar with the reproductive system organs’ names from overhearing the women in their community discussing their SRH problems. The only married girl in the FGDs was in her second pregnancy trimester during her participation and the only information she knew was that the fetus “goes out of the uterus”. Very few participants were able to identify the different organs of the female reproductive systems and their functions. Those participants received information through lectures offered by one local and another international NGO, in addition to biology classes. However, the participants expressed their preference to receive information through the lectures at NGOs because they were clear and explained using illustrations, whereas the information presented in biology classes focused only on mammals’ female reproductive system, to which participants could not relate:

“*It is not the same in biology class. The teacher talks about animals in the class, but we talk more about ourselves and look at illustrations at the institute*.” (14 years old).

### The need for accurate information on SRH topics

Throughout the FGDs, girls mentioned different sources from which they received information about SRH topics such as puberty, menstruation, and female physiology. The level of access to sources varied significantly from one participant to another. Mothers were the point of reference mentioned most frequently across all the FGDs. The girls stated other sources, such as female relatives, friends, local and international NGOs, the internet, biology classes, and teachers. A few participants chose not to ask for information, because they found the process embarrassing. Instead, they counted on self-made conclusions from overhearing and observing others. However, the participants indicated their wish to be able to speak to a specialized practitioner:

*“It would be good to have a center where a woman teaches us about the body changes. Those are very useful information.”* (17 years old).

*“I talk to my mom, but I would prefer to talk to a doctor who is specialized in such issues.”* (14 years old).

According to the girls, the exchange of knowledge should be done in a comfortable and private atmosphere with a trust-worthy person, who is direct and easy to talk to. Some participants expressed their concerns regarding their lack of knowledge about future SRH issues and experiences such as body changes and sexual intercourse. They showed interest in learning more about these topics:

*“I would prefer someone to talk to me directly about sexual intercourse so that I don’t get scared when it happens. It is something upcoming and I am scared about it now.”* (16 years old).

### Sexual harassment experiences and handling mechanisms

#### Shared experiences of sexual harassment

All FGDs participants reported that either they themselves, or a girl they knew, had been subjected to sexual harassment by a fellow male refugee or a male from the host community. They described their experiences as shared and ordinary incidents since they happened often:

*“I was sexually harassed many times when I was in school. That’s why I left school”.* (15 years old).

*“I’ve experienced a lot of harassment. We are used to it now. It has been like this ever since we arrived here.”* (17 years old).

Verbal sexual harassment experiences included ‘cat calls’ (public sexually indicative requests or remarks), comments on the girls’ clothing or body, and asking for certain favors:

*“It happened with a friend of mine. We were walking on the street. Some guys started cat calling, commenting on what my friend was wearing and the look of her hair.”* (14 years old).

*“Once a guy was following me with his car. He winked at me and asked me to join him in the car. I told him that I don’t want to and changed my direction.”* (17 years old).

Physical sexual harassment incidents involved unwanted touching of the body and clothing.

*“A friend went to the supermarket to buy pads for her sister. The guy thought that they were for herself. He touched her. She tried to avoid him, but he insisted. She ran out of the shop while crying.”* (17 years old).

*“I was getting food for my family. An old man followed me. He tried to touch me, but I ran away and went back home.”* (14 years old).

Non-verbal sexual harassment events included frequent following, whistling, staring, and surveying the girls’ bodies:

*“It happens to me whenever I go to school. Last time, a guy followed me with his car. I had an umbrella with me, I thought that I would hit him if he approached me.”* (14 years old).

*“A guy follows me whenever I go to my friend’s place. I go inside a building to hide. I still find him there when I go out. I change my direction and then go to my friend.”* (14 years old).

Most girls did not inform their parents about their experiences. During the FGDs, the girls frequently talked about their parents’ lack of understanding and the fear of being blamed. Additionally, the participants shared their anxiety that their parents would discover what happened to them, which would lead to them being subjected to mobility restrictions and made to drop out of school:

*“I feel embarrassed to tell my parents, they will not allow me to go to school anymore.”* (14 years old).

*“My friend was sexually harassed, but she did not tell her parents; she feared them. They will think that she caused it.”* (13 years old).

*“We are used to not sharing what happens to us with others. They would not let us go out after that. That’s the mentality of parents*.” (16 years old).

Despite the fact that they considered their mothers more tolerant compared to their fathers, very few participants reported sexual harassment incidents to their mothers. Two participants complained about their mothers’ negligence on the issue after informing them about their experiences.

#### Psychological effects of sexual harassment

The girls expressed their feelings of worry and fear about being sexually harassed when visiting public spaces. They talked about the offensive acts of being subjected to assault, privacy intrusion, and disregard for personal will:

*“I feel scared and nervous when someone follows me. I think that he will harm me.”* (14 years old).

*“We take the bad example from the bad guys. When I go with my sister to buy something from the shop in the evening, my mom keeps on watching us from the balcony, and we get really scared if we see a guy on the street. We start to run.”* (13 years old).

A few participants claimed no nervousness concerning sexual harassment, since they are always ready to protect themselves:

*“I am not scared of it because I know that I can defend myself. I can reply to the guy if he cat calls.”* (13 years old).

#### Strategies to deal with sexual harassment

The participants elaborated on approaches to avoid and respond to all forms of sexual harassment. Girls’ empowerment; good parenting and boys raising; physical self-defense; ‘appropriate’ girls’ behavior; asking for adult help; ignorance; getting people’s attention; and not going to public spaces alone were the tactics suggested by the girls:

*“Every girl should have a strong personality to defend herself. She should not be silent about it.”* (15 years old).

*“Parents should take care of their boys and check how they are behaving. Some parents don’t do that.”* (13 years old).

*“We should not go into small streets. We should walk on the main street where a lot of people are present, because he might push you into a building.” (*15 years old).

The adolescent girls expressed their wish to be able to report the incidents to an adult they trust and can freely talk to without being accused, as a method to protect themselves from the harmful consequences of sexual harassment. Parents, close relatives, policemen, and NGO female workers were the actors suggested by the participants from whom they wish to seek help.

*“Every girl needs someone to be at her side and defend her. She needs to know that she is safe”.* (14 years old).

## Discussion

This qualitative study is one of very few exploring SRH perceptions and experiences among Arab and Kurdish Syrian adolescent refugee girls in an urban setting in Lebanon. Its findings present important outlooks on the girls’ perceptions, experiences, knowledge, practices, and concerns regarding their SRH.

Before representing and tackling the different findings of this study, it is salient to contextualize them in alignment with the challenging legal and financial status of Syrian refugees living in Lebanon, the worsening Lebanese political and economic circumstances, and the inequitable healthcare system in the country. Lebanon did not sign the 1951 UN convention or its 1967 protocol concerning refugees’ status and its administration. The state signed instead a Memorandum of Understanding (MoU) with UNHCR in 2003, which defines the country as a place of transit and not of asylum. This gives the Lebanese government the authority to establish the legal status of refugees, which determines their rights, in line with its own laws (61, 62). Since 2014, many policies have been applied in Lebanon aiming to limit the number of Syrian refugees in the country. Thus, an increase in informality and illegality in addition to deteriorating living conditions has arisen (63). At present, 78% of Syrian refugees do not acquire legal residency, which restrains their freedom of movement, capability to work legally, and access to services such as healthcare and education (64, 65). Additionally, the Lebanese Ministry of Labor allows Syrian refugees to work in only three sectors: construction, agriculture, and cleaning (63). As a result of the legal restrictions and the economic crisis that Lebanon has been experiencing since 2019, it is estimated that at least 75% of Syrians in Lebanon live below the poverty line (66, 67).

Lebanon received a large number of refugees; however, the limited resources and services, in addition to poor infrastructure, has generated tensions between Lebanese nationals and Syrian refugees (12). Furthermore, the economic and political crises in the country challenge intercommunal social cohesion (68). Several reports highlight concerns regarding low social cohesion between Lebanese and Syrian refugees (69–71). Social cohesion is important for improving both individual and public health (72, 73). The Lebanese healthcare system is considered inequitable with a fragile structure that mainly concentrates on delivering health services, without taking into account the relevance of prevention, design, availability and accessibility of services (74, 75). Such a system produces health inequalities, where differences in socioeconomic status, gender, race or ethnicity between distinct groups in the country generate disproportion in terms of morbidity, mortality, and access to health services (76–78). The public healthcare system in Lebanon was difficult to access and afford even before Syrian refugees’ arrival (75). However, with the influx of a significant number of Syrian refugees, the system has become further strained and refugees struggle to receive sufficient health services (75).

The results of our study show that Syrian refugee adolescent girls have inadequate information on SRH issues. The majority of participating girls did not have any knowledge about either menstruation or the physical changes associated with puberty before experiencing them. The lack of preparatory understanding made them go through stressful and frightening experiences. Similar results were found among very young adolescents displaced from Myanmar living in Thailand, where only one in three girls reported being knowledgeable about changes during puberty before they happened (16). Migrant and refugee women from various cultural groups who have resettled in Australia and Canada stated that they had no knowledge of menstruation before menarche and that they became aware of the role of menstruation only after their pregnancy (79).

According to the program of action adopted at the ICPD, one essential component of SRH is the individual right to obtain sufficient knowledge on sexual health (21). The SRH literacy status of an individual is affected by cultural, religious, and social factors in addition to the quality of the accessible healthcare facilities and services (80, 81); however, SRH literacy is mainly built through education and factual scientific knowledge. SRH literacy allows women to build up behavioral, existential, cognitive, and advocacy competences that lead to the decrease of gender and health inequalities concerning SRH (82–84). Adolescence is a sensitive phase accompanied by rapid physical and emotional changes and the mandate of new social roles, which can put adolescents under stress. However, a distinct vulnerability is experienced by adolescents in cases of forced displacement (85). The adolescent girls in this study talked about their particularly distressing experiences after reaching puberty. A qualitative study on the health of very young Syrian adolescents living in an urban and an ITS setting in the Lebanese Governorate of Bekaa showed that the participants had a poor comprehension of puberty (86). All adolescents worldwide encounter challenges when seeking information on SRH issues, but adolescent refugees’ experiences of displacement create additional obstacles when searching and acquiring SRH knowledge (87). Interrupted education, divided families, incidents and extortions of sexual violence, and inadequate access to healthcare systems affect refugee adolescents’ SRH literacy (87).

Adolescent girls in this study mentioned three kinds of accessible sources of SRH knowledge: individuals (e.g. mothers, female relatives, friends); institutions (e.g. schools, local and international NGOs); and media (e.g. YouTube). Similar to studies in other humanitarian settings in different regions, mothers were the main source of SRH information for adolescents (16, 88). However, the girls expressed their interest in having access to additional knowledge from experts in the field of SRH in addition to their worries about not receiving sex education. Peers, media, and other informal sources are the predominant sources for SRH knowledge for young people, which enable the sharing of incorrect information and the generation of misconceptions (87, 89–91). The Syrian refugee girls who participated in this study had fewer opportunities to access information about SRH, since nearly 43% of them are experiencing disrupted schooling and 55% of them have never participated in NGO activities or training. Adolescent girls described myths concerning beneficial and harmful practices during menstruation in addition to traditions that help in controlling body changes during puberty. The misconceptions mentioned are the result of present cultural beliefs in the girls’ communities or their misinterpretation of incomplete information, since they described feeling embarrassed to ask for information on SRH issues and their preference instead for self-made conclusions.

Our findings indicate the urgent need for accessible programs in Lebanon that deliver SRH knowledge to Syrian adolescents in general and Syrian girls in particular. However, this finding is most likely also transferable to other recipient countries in the region, including Lebanon, where adolescent girls’ SRH and puberty experiences are overlooked in research (92). The scientific information should be presented in a clear, simple, and comprehensive way. The participants in this study who had the opportunity to participate in NGOs and scouts’ educational events showed satisfactory knowledge about different SRH topics. They talked about their positive experiences receiving direct information that was explained and demonstrated to them via illustrations. Comprehensive sex education allows adolescents to acquire fundamental information about SRH issues and to develop decision-making skills that are essential when dealing with those issues (93). Schools are a key formal source for SRH knowledge, where students receive the information within a complete learning setting (79). In Lebanon, students learn partially about sex education as part of the biology curriculum (94). However, Lebanese adolescents, girls and boys equally, expressed their wish to receive a complete reproductive health education (95). A study demonstrated that Lebanese adolescents who received school lessons on reproductive health, showed more careful practices during their first sexual experience (96). A 2019 vulnerability assessment of Syrian refugees in Lebanon found that 69% of primary-school age Syrian children were enrolled in schools, whereas only 22% of secondary-school age Syrian children were enrolled. The main reasons for school dropouts were cost-related burdens, child labor, and marriage (65). It is essential to recognize these barriers when implementing humanitarian educational programs in order to make them accessible for adolescent refugees and to meet their SRH needs. The emotional health and socioeconomic factors that affect the everyday lives of Syrian adolescent girls in Lebanon should also be taken into account when designing intervention plans.

This study also shows the important role of mothers as an accessible and trusted source of SRH knowledge for adolescent girls. Thus, this existing key role should be acknowledged and developed by the working NGOs to be more effective. Firstly, it is necessary to provide refugee mothers with concrete information about SRH issues to share with their daughters and prevent them from sharing misconceptions. Secondly, it is fundamental to actively engage them in the learning process through their inclusion in the development and employment of educational programs offered by the different existing organizations. Such an approach with two phases of measures will encourage the mothers to start discussions on SRH topics with their daughters, with the accurate information needed. Previous studies have shown that refugee mothers in general and Syrian refugee mothers in Lebanon in particular express concern about not having adequate information about SRH subjects that they can deliver to their daughters. The mothers also request the facilitation of their education in order to be equipped with knowledge about SRH issues and to be able to communicate in a culturally suitable way with their adolescents (79, 86, 20). Social and cultural customs that consider conversations on SRH issues a taboo are the main obstacle for SRH literacy (90, 91, 97, 98). Syrian refugees in Lebanon are diverse, coming from various rural and urban regions in Syria, with different levels of education and cultural practices (20). This diversity should be recognized by the working actors in the humanitarian field, who should not perceive Syrian refugee girls and women as a homogenous group when designing and implementing intervention programs. Instead, they should provide them with knowledge that complements their individual learning and health needs. Plan International recommends working actors and service providers in Beirut and its suburbs collaborate in implementing programs that tackle the specific needs of adolescent girls, with the prioritization of the most vulnerable ones, in order to prevent duplication of interventions in some fields and neglect for other fields (12). Plan International also suggests designing intervention programs based on needs assessments that provide segregated data by sex, age, and disability. Reaching refugee communities in an urban setting is difficult. Therefore, there is a need to expand community outreach and awareness campaigns that inform adolescent refugee girls about available programs and services (12).

The participants reported their frequent exposure to different types of sexual harassment by fellow male refugees or males from the host community. The girls were harassed when in public areas, mainly in the streets, which they described as ‘becoming ordinary’. The consistent harassment caused the girls to feel anxiety and fear, resulting in school absenteeism for some of them. Similar results were found among Syrian adolescent girls and young women living in a neighborhood in Izmir, Turkey, where displacement and changed social circumstances increased their exposure to violence and mainly to verbal, sexual, and physical street harassment (99). Previous studies done in Lebanon on the same issue showed that Syrian parents limited their adolescent daughters’ mobility when unaccompanied due to safety concerns. In order to protect them from possible public sexual harassment and assaults, parents implemented strategies that had a negative impact on their adolescent girls, such as dropping out of school and child marriage (100, 101). Roupetz et al. (101) describe this as a continuous cycle of SGBV where some parents replace one type of violence with another. This also might explain the feeling of victimization among the participants in our study when reporting harassment to their parents, and their preference to endure harassment in order to attend school and have partial freedom of movement.

After displacement to a host country, women and girls often encounter social isolation and extreme financial need, which make them more vulnerable to sexual exploitation and assaults (102, 103). In addition to that, they face discriminations because of their nationality, ethnicity, race, language, class, gender, sexual orientation, and physical disability (15). It is key to recognize the complexity of urban settings and the types of risks present in them in order to design effective SGBV intervention programs. In its report “Mean Streets: Identifying and Responding to Urban Refugees’ Risks of Gender-Based Violence”, the Women’s Refugee Commission identifies the common SGBV risks in four urban settings, including Beirut, which are associated to: livelihoods, shelter, urban isolation, fear of the police, and lack of access to justice (15). A study conducted in three different locations in Beirut, including Bourj Hammoud, found that migration status plays a major role in forming adolescent girls’ attitudes towards the neighborhoods they live in and their feeling of safety there: 87% of Lebanese girls felt welcomed in their neighborhood whereas only 65% of Syrian girls felt the same (12). Finally, research and intervention programs should not overlook the composite and multilayered experiences of violence lived by Syrian refugee women in Lebanon by focusing only on gender relevant indicators (75). As Yasmine and Moughalian (2016) explain, microsystem (an individual’s direct context and communication), exosystem (all types of institutions affecting an individual’s behavior), and macrosystem (cultural perceptions and approaches) determinants should be closely examined to understand their effects on Syrian refugee women’s SRH status in Lebanon. By adapting the social ecological model, Yasmine and Moughalian (2016) show that the conflicts occurring and pressures that exist related to Syrian refugee women’s agency, along with Lebanese institutional constrictions, are the result of gendered roles and assumptions founded upon the micro-, exo-, and macro-systems’ numerous layers (75).

In a very dense urban context like Bourj Hammoud, the high variation of Syrian refugees’ legal status and therefore the legal precarity of their families was noted, which has a decisive health impact on adolescent refugee girls and young women. This very significant factor is noted and will be further investigated in a future publication.

## Study Limitations

The study faced some challenges and limitations. Parents’ disapproval of the research topic and their daughters’ participation hindered access to the girls. SRH is a sensitive issue that is not welcomed for open discussions in the local community. Safety concerns in general and fear of sexual violence in particular may have restricted mobility for some girls to participate in the FGDs. Some refugee parents in Bourj Hammoud do not allow their daughters to be left unaccompanied when leaving the apartment or participating in an activity. This made access to Syrian refugee adolescent girls more difficult. During the FGDs, social pressure may have discouraged some participants from sharing their personal opinions and experiences on SRH, specially on more sensitive topics such as domestic and intrafamily violence. Furthermore, participants might have chosen specific answers that they thought the moderator or peers preferred. Finally, collecting data on such sensitive topic using multiple qualitative methods- such as observation, body mapping, and interviews- in different Lebanese urban contexts that are inhabited by refugees, would have given this study a deeper understanding of the refugee adolescent girls’ SRH. However, this was not possible for our study due to capacity constraints of the study team when in the field in Lebanon.

## Conclusions

This study provides insights on the SRH of Syrian adolescent girls living in an urban setting in Lebanon. Its findings present an understanding of the girls’ perceptions, experiences, knowledge, practices, and concerns regarding their SRH. The girls appear to be in need of solid information about SRH issues, for example through accessible programs. Additionally, the role of mothers as trusted and accessible sources of information should be recognized and supported by program developers in the field. The frequent exposure of girls to street sexual harassment should be understood within the complex setting of an urban area, along with the SGBV risks it presents, in order to offer interventions that reduce SGBV incidents.

## Supplementary Information

**Additional file 1.** Focus Groups Discussions Guide (45 min-1 h).

**Additional file 2.** A Sample of the Codebook’s Schematic Representation.

## Data Availability

The dataset and materials used in this study are available from the first author on reasonable request.

## Abbreviations

ASRH: Adolescent Sexual and Reproductive Health
ASRHR: Adolescent Sexual and Reproductive Health and Rights
FGD: Focus Group Discussion
EMENA: Extended Middle East and North Africa
IAFM: Inter-Agency Field Manual on Reproductive Health in Humanitarian Settings
IAWG: Inter-Agency Working Group on Reproductive Health in Crises
ICPD: International Conference on Population Development, ITSs: Informal Tented Settlements
LCRP: Lebanon Crisis Response Plan
MHM: Menstruation Hygiene Management
MoU: Memorandum of Understanding
NGO: Non-governmental organizations
SGBV: Sexual and Gender-based Violence
SRH: Sexual and Reproductive Health
SRHR: Sexual and Reproductive Health and Rights
UNFPA: United Nations Population Fund
UNHCR: United Nations High Commissioner for Refugees
