# Supplementary material for: Sexual and reproductive health of Syrian refugee adolescent girls: a qualitative study using focus group discussions in an urban setting in Lebanon
Source: Reprod Health. 2021 Jun 24;18:130. doi: 10.1186/s12978-021-01178-9 (PMC8223310; doi:10.1186/s12978-021-01178-9)
Supplement: Supplementary file 1 — Additional file 1. Focus Groups Discussions Guide (45 min-1 h). [file 12978_2021_1178_MOESM1_ESM.pdf]

Additional File 1

Focus Groups Discussions Guide (45 min-1 h)

Instructions: FGDs will be done with Syrian refugee young girls living in Bourj Hammoud within the age group of 13-17 years. Every FGD will be conducted with 5 participants. The moderator will be responsible of asking the questions and keeping the conversation within the different themes. The student assistant will be responsible of taking notes on nonverbal communication and recording the discussion. Both the moderator and the assistant should introduce themselves and move to the introduction.

Background information on FGD participants: age, marital status, ethnic group, governate of origin in Syria, date of arrival to Lebanon, highest achieved level of education, if currently enrolled in school, if currently participating in NGOs activities/trainings.

Theme 1: Menstruation

1. Have you had your menstruation? What was your experience upon menarche? How do you perceive it now?
2. Did anyone explain to you what menstruation is or why it occurs before having it? With whom do you talk about you experience? Is your day affected when you get your period (pain, social and physical activities, hygiene…)?
3. Do you know how to track it?
4. What materials do you use during your menstruation? How do you get access to them?

Theme 2: Puberty

1. What is puberty? How do you define it? At what age is it reached by girls?
2. What are the changes that happen to a girl's body during puberty? What do you think of them?
3. What was/is your experience during puberty?

Theme 3: Sexual and reproductive health awareness

1. How do you define sexual and reproductive health?

This question was changed later to: how do you define adolescent girls' health?

2. Whom do you approach if you have any question regarding your body? Do you prefer to talk to someone else? Why?
3. Did you ever receive information about the reproductive systems of men and women and their functions? Can you name some of them? What are their functions?
4. If you need SRH services, whom do you ask? where do you prefer to go? To whom do you prefer to talk?

Theme 4: Sexual Harassment

1. Were you or do you know a girl who was subjected to sexual harassment while living in this area? How did it happen?
2. What did you/she do after the incident (did she talk to someone/reported it/sought help)?
3. What are the things you think might be of help to cope with and prevent experiences of sexual harassment and other forms of SGBV?
