# Supplementary figures and images for: Sexual and reproductive health of Syrian refugee adolescent girls: a qualitative study using focus group discussions in an urban setting in Lebanon
Source: Reprod Health. 2021 Jun 24;18:130. doi: 10.1186/s12978-021-01178-9 (PMC8223310; doi:10.1186/s12978-021-01178-9)

## Additional File 2

### A Sample of the Codebook's Schematic Representation

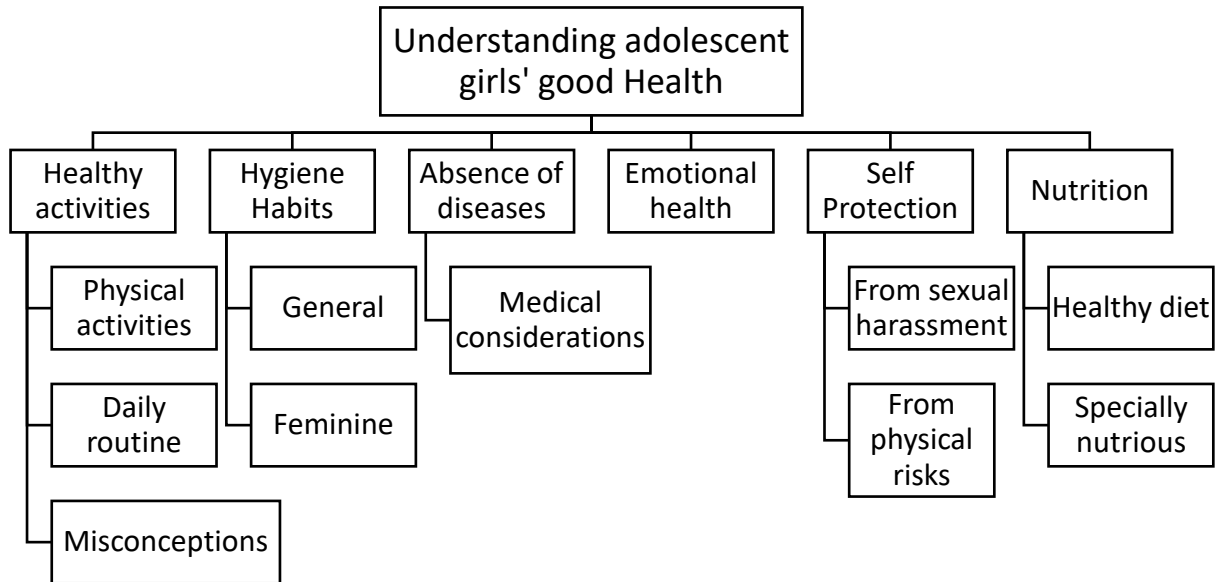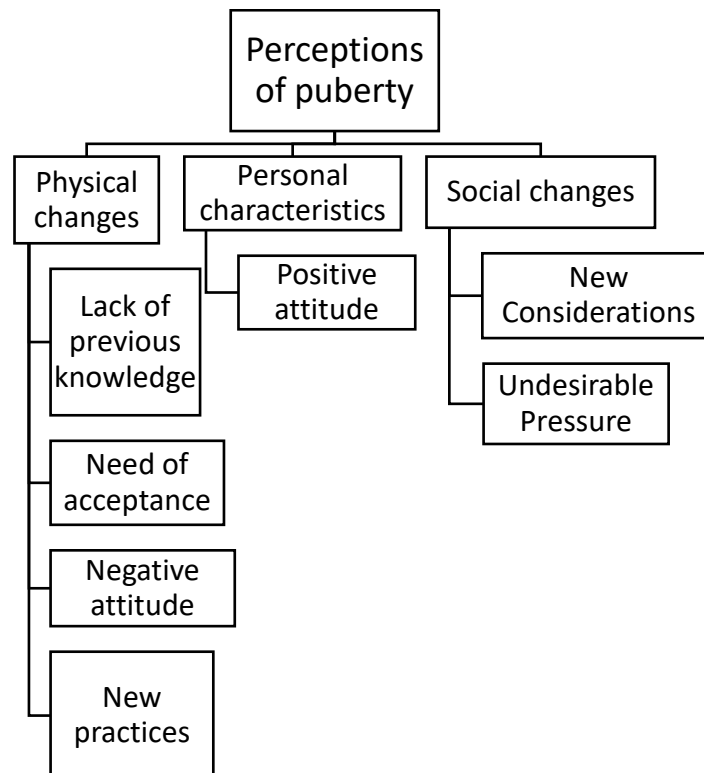

Supplement: Supplementary file 2 — Additional file 2. A Sample of the Codebook’s Schematic Representation. [file 12978_2021_1178_MOESM2_ESM.pdf]
